# Ventriculus terminalis cyst in an infant: a case report

**DOI:** 10.1186/s13256-023-03759-7

**Published:** 2023-01-23

**Authors:** Arnold H. Menezes, Yutaka Sato, Brian J. Dlouhy, Karra A. Jones, Steven A. Moore

**Affiliations:** 1 Department of Neurosurgery, University of Iowa Hospitals & Clinics and Stead Family Children’s Hospital, Iowa City, IA USA; 2 Department of Radiology, University of Iowa Hospitals & Clinics and Stead Family Children’s Hospital, Iowa City, IA USA; 3 Department of Pathology, University of Iowa Hospitals & Clinics and Stead Family Children’s Hospital, Iowa City, IA USA; 4 Department of Neurosurgery, University of Iowa Hospitals & Clinics, 200 Hawkins Drive, 1824 JPP, Iowa City, IA 52242 USA

**Keywords:** Case report, Extra-axial cauda equina cyst, Filar cyst, Lipomatous filum, Tethered cord, Ventriculus terminalis

## Abstract

**Background:**

Filar cysts are frequently found on neonatal ultrasound and are physiologically involuting structures with natural resolution. Hence, there has been no previous histologic correlation. Ventriculus terminalis is a focal central canal dilation in the conus medullaris and usually not clinically significant. Extra-axial cyst at the conus–filum junction connected to ventriculus terminalis is extremely rare, especially when associated with tethered lipomatous filum terminale and with progressive cyst enlargement.

**Case presentation:**

A Caucasian female neonate with abnormal gluteal cleft had ventriculus terminalis cyst with an extra-axial cyst at the conus–filar junction and taut lipomatous filum on ultrasound examination and magnetic resonance imaging. This persisted at 6-month follow up imaging. In light of the nonresolving extra-axial mass and thick taut lipomatous filum, the child underwent L1–L3 osteoplastic laminectomies. The extra-axial cyst expanded after bony decompression and furthermore on dural opening; visualized on ultrasound. It communicated with the central canal and was documented with intraoperative photomicrographs. It was excised and filum sectioned. Histological immunostaining of the cyst wall showed neuroglial and axonal elements. The child did well without deficits at 4-year follow up with normal urodynamics.

**Conclusion:**

Progression dilation of ventriculus terminalis and extra-axial conofilar cyst with tethered lipomatous filum will likely progress to clinical significance and require surgical intervention. The embryologic basis for this pathology is discussed, with literature review.

## Introduction

Incidental cystic mass at the conofilar junction is frequently found at the neonatal lumbar ultrasound (US) examination and termed as filar cyst (FC). FC is considered a physiologic involuting structure with expected natural resolution [1, 2]. The ventriculus terminalis (VT), a focal dilation of the central canal in the conus medullaris is also relatively frequently identified at ultrasound and magnetic resonance imaging (MRI) of neonates and young infants. The majority of ventriculus terminalis regress before 5 years of age and are also considered as the transitional structure [3]. We present a case of a 6-month-old infant with a nonresolving expanding conofilar junction extra-axial cyst that had communication with the central canal of the conus medullaris. Also found at the surgery was the tethering of the cord to the thick filum with fibrolipoma. Some conofilar cyst masses when associated with other distal cord anomalies such as tethered cord warrant closer observation, clinically and by imaging, for the appearance of cauda equina symptoms and progressive enlargement. Surgical intervention may be necessary.

## Case report

**Fig. 1:**
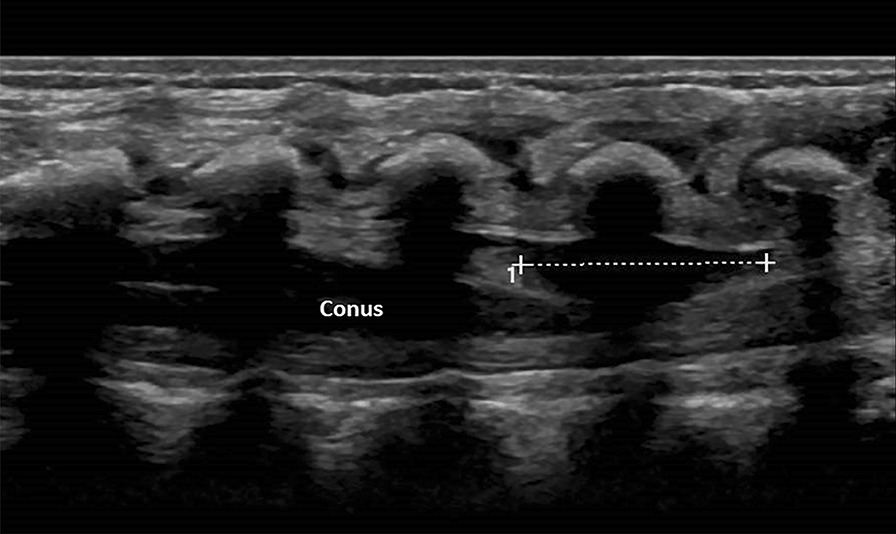
Sagittal ultrasound of lumbar spine at 1 day of age. The conus ends at L2. Note the oblong anechoic cyst at the conofilar junction dorsal to the conus medullaris

**Fig. 2:**
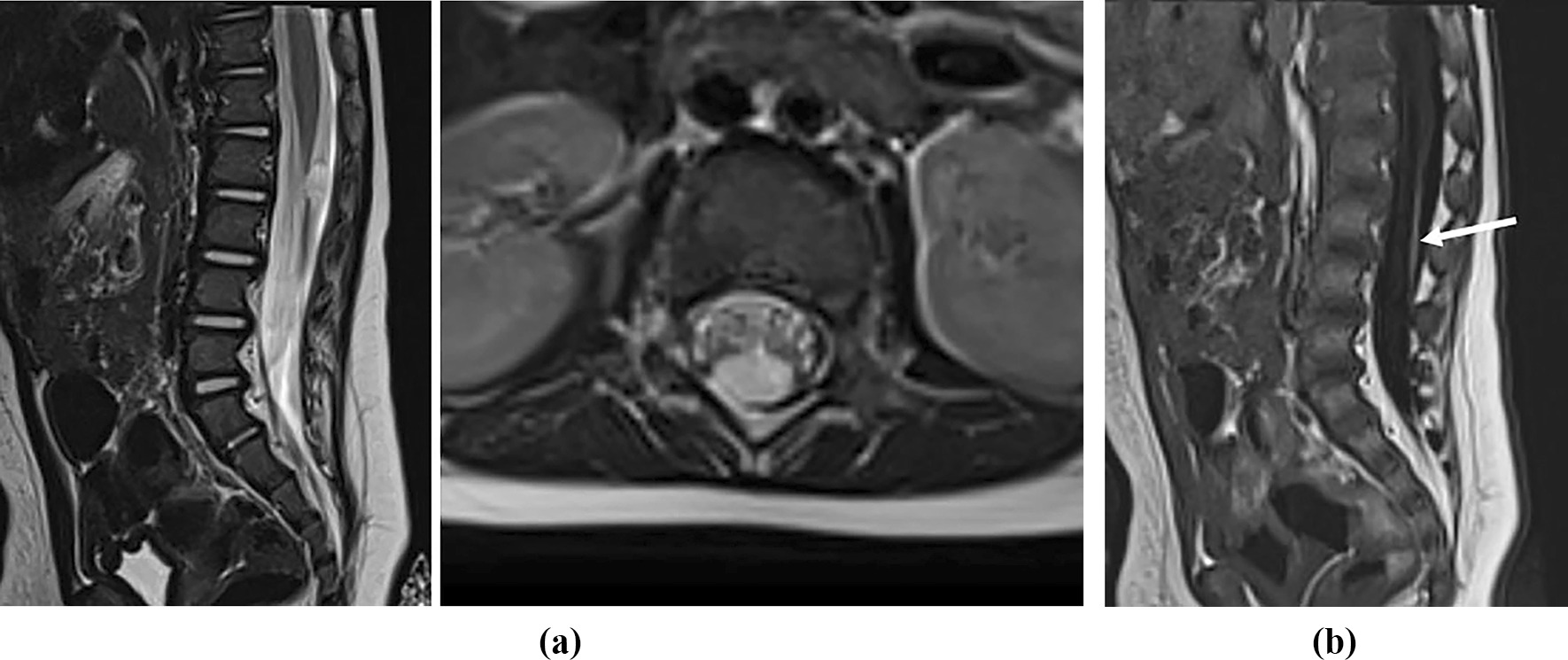
**A** Midsagittal and axial T2-W magnetic resonance image of lumbar spine at 6 months of age visualizes the persistent conofilar cyst. **B** Midsagittal T1-W magnetic resonance image at 6 months of age demonstrates a thick filum with fibrolipoma (arrow)

A neonate Caucasian girl, a product of an uncomplicated pregnancy, was found to have asymmetric gluteal crease. Neurological examination was normal, and subsequent urodynamics study was also normal. Spine ultrasound at 1 day of age showed a cystic mass overlying the conofilar junction at the L3 level measuring 12 × 5 × 5 mm (Fig. 1). The termination of the conus medullaris was at L2. MRI showed the cystic mass dorsal to the conus emanating at the conofilar junction (not shown). A follow-up MRI at 6 months continued to show the cystic mass and a fibrolipoma of the filum terminale (Fig. 2A, B). This study was approved by the institutional review board, and parental consent was obtained. Written informed consent was obtained from the patient’s legal guardian for publication of this case report and its accompanying images. A copy of the written consent is available for review by the Editor-in-Chief of this journal.

**Fig. 3:**
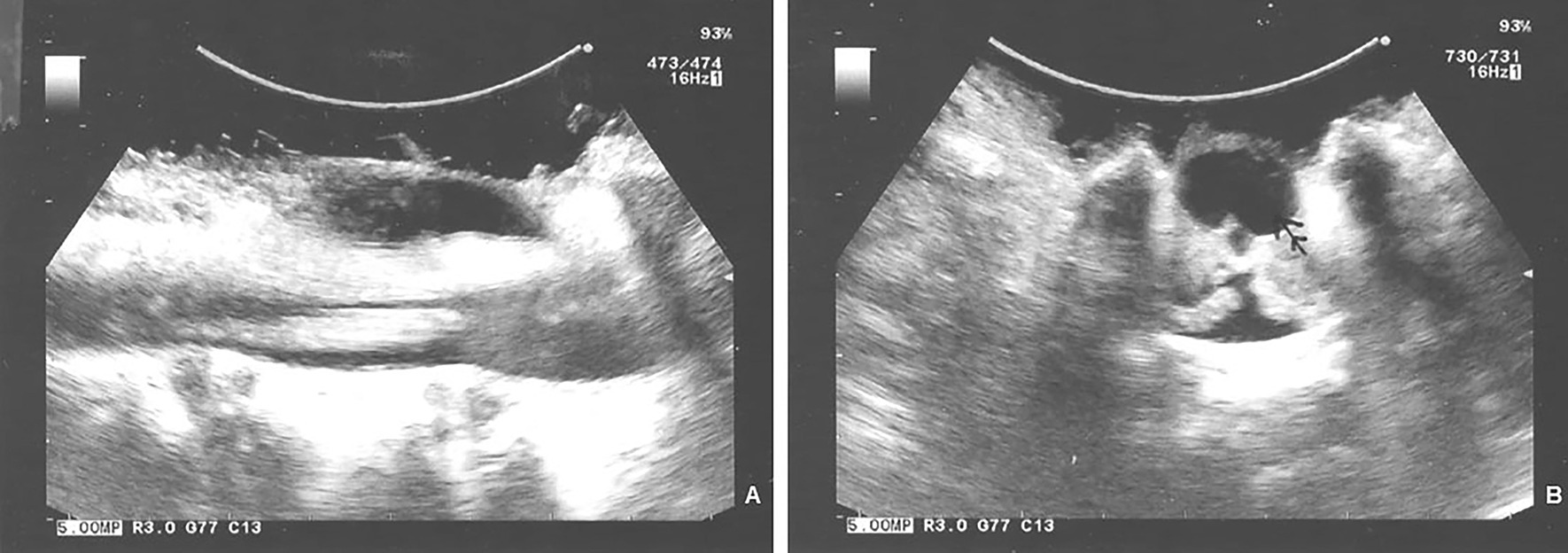
Sagittal (**A**) and axial (**B**) intraoperative ultrasound after L1–L3 laminectomies with intact dura. The cyst (arrow) appears to be larger compared with the size demonstrated on magnetic resonance imaging

**Fig. 4:**
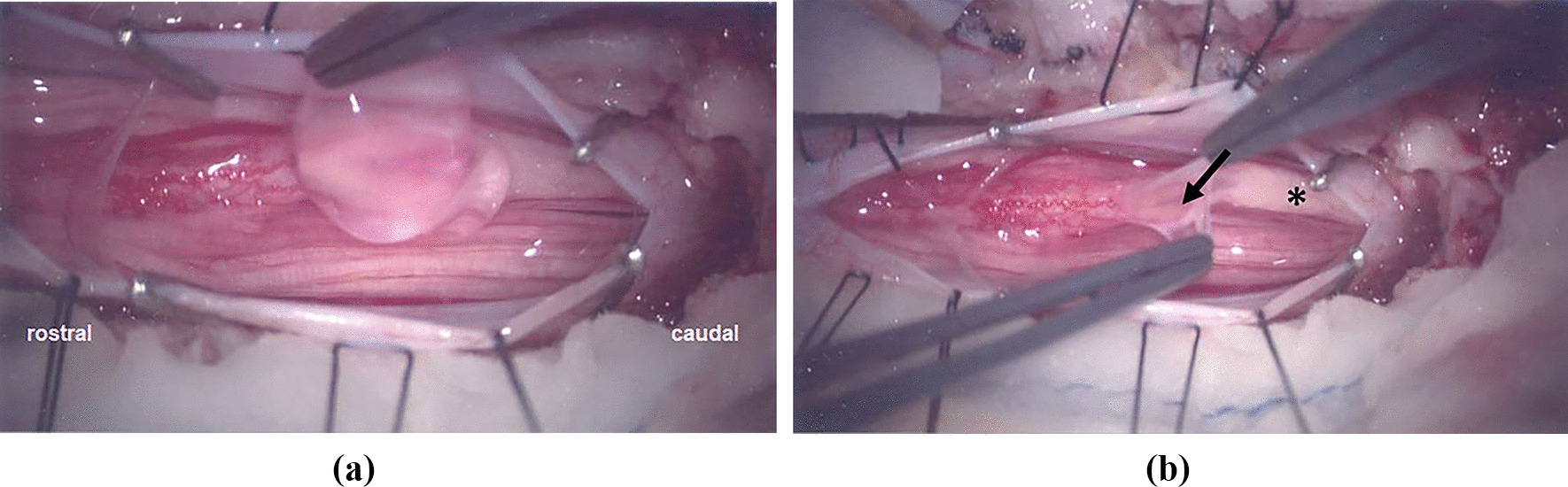
**A** Intraoperative photograph through the microscope. Upon opening the dura, the cyst further expanded and “mushroomed out” into the operative field. **B** The cyst is opened and communicates with the central canal of the conus (arrow). The thick lipomatous filum is seen (*)

In light of the nonresolving extra-axial cystic mass and the thick lipomatous filum, the patient underwent L1–L3 osteoplastic laminectomies. Intraoperative ultrasound prior to opening the dura confirmed the cyst (Fig. 3), which appeared to be larger than that seen on the preoperative MRI. The filum was thick and hyperechoic, and the spinal cord demonstrated no motion with cardiac or respiratory cycle confirming the cord tethering. Upon the opening of the dura, the cyst, which was apparently under pressure expanded further, in a “mushroom” fashion (Fig. 4A). The cyst was opened, and clear cerebrospinal fluid (CSF) gushed out. There was a communication channel between the cyst lumen and the central canal of the conus (Fig. 4B). The cyst wall was excised, and the filum was sectioned 2 cm below the conofilar junction. Pathologic evaluation of the cyst wall demonstrated both neuroglial and fibrous tissues. Immunoperoxidase staining confirmed that both glial and axonal elements were present. Postoperatively the patient remained free of neurologic deficits.

At 3-year follow up the child had a normal neurological examination and was walking well. She was bladder toilet trained.

## Discussion

On neonatal lumbar spine sonography, a simple cyst at the conofilar junction is frequently observed and termed as a filar cyst [1]. These cystic structures are midline in location, inferior to the conus, thin walled, anechoic, and fusiform on longitudinal and round on transverse plane on ultrasound. The majority of filar cyst regress. Because of a lack of histologic correlation, the exact pathology of filar cyst is not clear. Irani *et al*. [1] found filar cyst in 12% of 644 spine US of neonates and infants. They consider that filar cyst is developmental in nature because the incidence of filar cyst detection was inversely related to age up to 6 months; there is a lack of filar cyst descriptions in adults. Thus, neonatal filar cysts incidentally found by ultrasound are considered as a normal variant. Seo *et al*. [2] followed 50 cases of filar cysts detected in neonatal ultrasound with subsequent MRI at age 5–12 months. Twenty filar cysts (40%) showed later regression, while 30 persisted and 2 showed progressive enlargement requiring surgery. It is noteworthy that these latter two cases had concurrent fibrolipoma of the filum and communication of the cyst with the central canal of the more rostral cord was observed during surgery.

During the first 5 years of life, incidental observation of a focal dilation of the central canal in the conus at lumbar ultrasound or MRI is frequent and termed as a ventriculus terminalis (VT). In their retrospective review of 418 spine MRIs (age 5 days to 20 years), Coleman *et al*. [3] found focal dilation of the central canal in the conus in 11 of 180 (6%) patients younger than 5 years but not among patients older than 5 years. Thus, asymptomatic localized terminal ventricle is considered as a normal developmental phenomenon. In adulthood, the persistence of the terminal ventricle, especially when unaccompanied by other central nervous system anomalies, is extremely rare. Symptomatic adult cases of enlarged terminal ventricle have been reported in surgical literature under the name of terminal ventricular cyst. The main presentations are either acute or chronic back pain, sciatica, bladder dysfunction, and/or lower extremity weakness with female predominance [4].

**Fig. 5:**
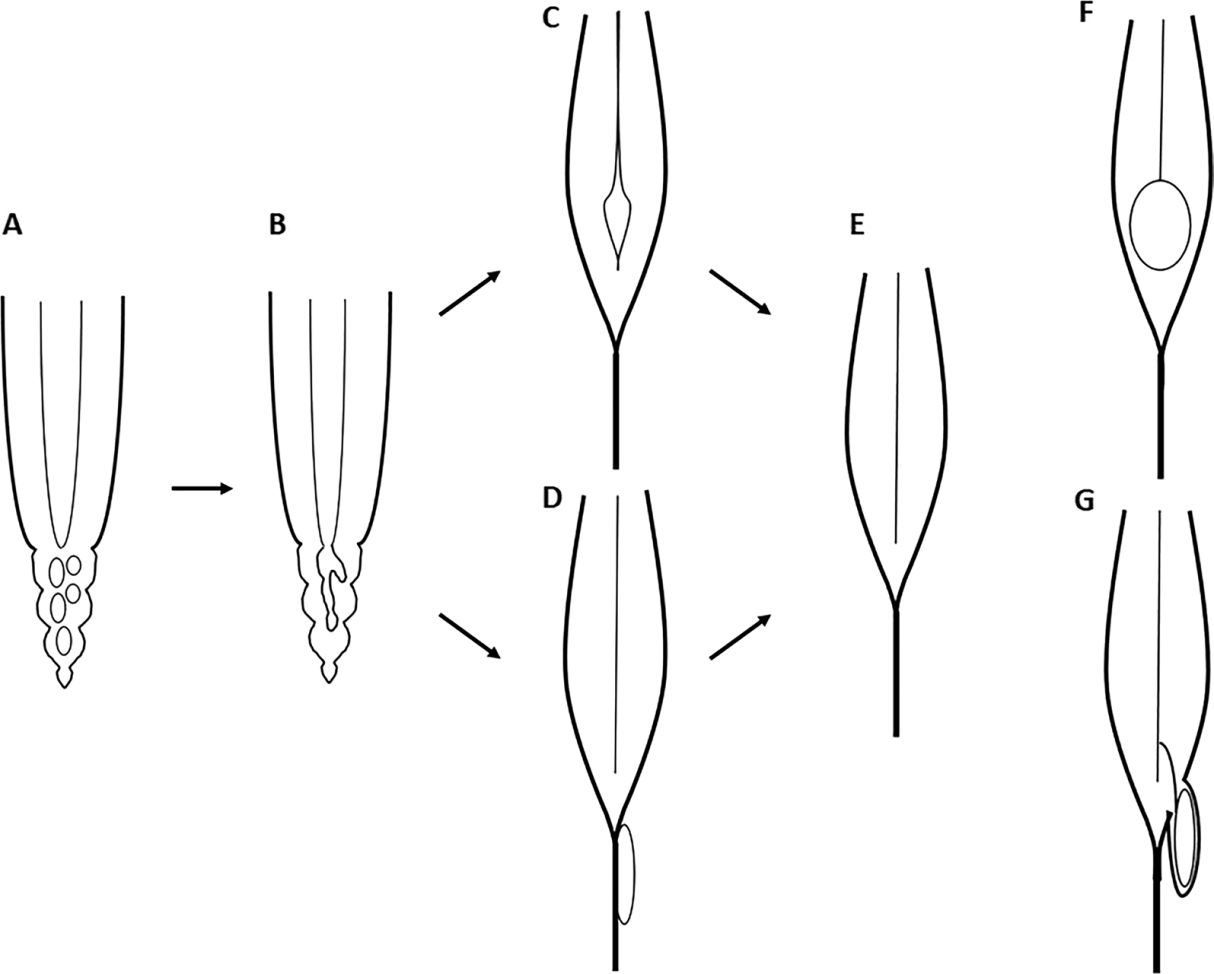
**A** Vacuolization of the equinal cord; **B** canalization with “forking”; **C** ventriculus terminalis; **D** filar cyst; **E** normally developed cord; **F** ventriculus terminalis cyst; **G** ventriculus terminalis cyst with “forking”

Are the ventriculus terminalis and filar cyst different entities or ends of the spectrum with the same pathophysiology? We believe the latter and these are representing the embryologic variation of the distal spinal cord (Fig. 5). Both the ventriculus terminalis and filar cyst are transitional structures in the equinal cord (the conus medullaris and filum terminale) during the process of retrogressive differentiation, which may be still in progress in early infancy [3, 5]. The process of the canalization and retrogressive differentiation of the equinal cord is extremely variable [5]. The filar cyst may represent residual of minor cystic loculation remaining in the equinal cord, which regress early in infancy. The ventriculus terminalis is a focal enlargement of the ependymal lined canal, in which the connection to the central canal of the more rostral spinal cord is maintained. If this connection is either narrowed, kinked or compressed by a mechanism such as cord tethering, deranged CSF flow dynamics may result in dilation of the ventriculus terminalis cavity with increased pressure. Central canal dilatation of the conus is well documented among patients with “tethered cord” such as with lipomyelocele, myelomeningocele, and diastematomyelia [6]. Alteration of the CSF dynamics in the central canal by the cord tethering was considered as a cause.

During the canalization of the equinal cord, vacuoles form at many sites and coalesce into the canal with great viability. The ependymal-lined cyst/canals in the conus are variable and may communicate with the central canal or remain as an isolated cavity [5, 7]. Accessory lateral and dorsal canals may extend into the filum and may communicate into the central canal. These variations are commonly seen in human embryos and referred as “forking.” Lendon and Emery evaluated 77 autopsy specimens of infants under 1 year of age and found the presence of the major “forking” in 10% and minor “forking” in 31% [7]. When “forking” of the canal is present in the conus, the ventriculus terminalis may be off the midline and may even mimic extramedullary location on the imaging, like in the case presented here.

## Conclusion

Ventriculus terminalis and filar cyst are transitional and should involute. Persistence and enlargement of associated extra-axial cysts and other equinal cord anomalies such as tethering of the cord warrants close observation for the development of cauda equina symptoms and progressive enlargement. Surgical intervention may be necessary.

## Data Availability

Data sharing not applicable to this article as no datasets were generated or analyzed during the current study.
